# Modelling Role of Protective and Nonprotective HLA Allele Inducing Different HIV Infection Outcomes

**DOI:** 10.1007/s11538-024-01334-9

**Published:** 2024-07-13

**Authors:** Shilian Xu

**Affiliations:** 1 Department of Environment and Genetics, School of Agriculture, Biomedicine and Environment, La Trobe University, Bundoora, VIC 3086 Australia; 2 Department of Mathematical and Physical Sciences, La Trobe University, Bundoora, VIC 3086 Australia

**Keywords:** CD8+ cell, HIV elite controller, HLA diversity, Bistable viral kinetics, HIV

## Abstract

Human immunodeficiency virus (HIV) infects CD4+ cells and causes progressive immune function failure, and CD8+ cells lyse infected CD4+ cell via recognising peptide presented by human leukocyte antigens (HLA). Variations in HLA allele lead to observed different HIV infection outcomes. Within-host HIV dynamics involves virus replication within infected cells and lysing of infected cells by CD8+ cells, but how variations in HLA alleles determine different infection outcomes was far from clear. Here, we used mathematical modelling and parameter inference with a new analysis of published virus inhibition assay data to estimate CD8+ cell lysing efficiency, and found that lysing efficiency fall in the gap between low bound (0.1–0.2 day^−1^ (Elemans et al. in PLoS Comput Biol 8(2):e1002381, 2012)) and upper boundary (6.5–8.4 day^−1^ (Wick et al. in J Virol 79(21):13579–13586, 2005)). Our outcomes indicate that both lysing efficiency and viral inoculum size jointly determine observed different infection outcomes. Low lysing rate associated with non-protective HLA alleles leads to monostable viral kinetic to high viral titre and oscillatory viral kinetics. High lysing rate associated with protective HLA alleles leads monostable viral kinetic to low viral titre and bistable viral kinetics; at a specific interval of CD8+ cell counts, small viral inoculum sizes are inhibited but not large viral inoculum sizes remain infectious. Further, with CD8+ cell recruitment, HIV kinetics always exhibit oscillatory kinetics, but lysing rate is negatively correlated with range of CD8+ cell count. Our finding highlights role of HLA allele determining different infection outcomes, thereby providing a potential mechanistic explanation for observed good and bad HIV infection outcomes induced by protective HLA allele.

## Introduction

Difference in disease outcome of autoimmune, bacterial infection and viral infection is strongly associated with diversity at human leukocyte antigens (HLA) alleles (Collins et al. 2020; Medhasi and Chantratita 2022; Dendrou et al. 2018). Protective HLA always lead to good outcomes while non-protective HLA correspond to disease progression (Collins et al. 2020; Mosaad 2015). HLA class I alleles presents a restricted set of HIV-derived peptides 8–11 amino acids in length, which determines antigenic specificity of CD8+ T cell responses (Collins et al. 2020; Medhasi and Chantratita 2022). CD8+ cells lyse infected CD4+ cells and release secrete antiviral cytokine via T cell receptors (TRC) on CD4+ cells (Migueles et al. 2015).

Human immunodeficiency virus (HIV) infects major human immune cells (including CD4+ T cells, macrophages, and dendritic cells) and then causes acquired immunodeficiency syndrome (AIDS) in which progressive failure of the immune system allows life-threatening opportunistic infections and cancers to thrive (Bekker et al. 2023). A small subset (approximately ~ 1%) of infected individuals, known as HIV elite controllers, can suppress viral replication with low or undetectable HIV viral load, strong HIV-1-specific CD8+ cell responses, and normal CD4+ cell counts without antiretroviral therapy (ART). A variety of research indicates that this durable control is mediated not by antibodies but by effective HIV-specific CD8+ cells (Pitisuttithum et al. 2006; Buchbinder et al. 2008; Rerks-Ngarm et al. 2009). For example, neutralizing antibodies do not mediate suppression of HIV Type 1 in elite controller (Migueles et al. 2000). Cellular immunity mediated by CD8+ cells can sustain long-term disease-free and transmission-free HIV control. Genetic polymorphisms of HLA class I alleles presenting viral peptides on the surface of infected cells for recognition by HIV-specific CD8+ T cells have shown to be associated with increased likelihood of control (protective HLA, for example, HLA-B57, HLA-B*27, HLA-B*52 and HLA-B*14) and others with risk of disease progression (non-protective HLA, for example, HLA-B*07, HLA-B*08 and HLA-B*35). Interestingly, being an elite controller is associated with having a protective HLA allele, but carriage of protective HLA alleles may not always confer patients good HIV control (Collins et al. 2020). This hints that different phenotypes of patients with protective HLA genotypes can be induced by multiple steady states of HIV kinetics. However, the mechanism by which a protective HLA allele leads to both good HIV and bad HIV control is still completely unclear.

In existing within-host models of infected cell and effector CD8+ cells interaction, CD8+ cell efficiency is assumed to be directly proportional to the product of both infected cell and CD8+ cell count (Cao et al. 2016; Ciupe et al. 2007; Goyal et al. 2019). CD8+ cells release granzymes and perforin to lyse infected CD4+ cells, but amount of granzymes and perforin carried by each CD8+ cell is limited. This indicates that for a given CD8+ cell count, its efficiency is supposed to increase but converge to its maximum with increase of infected cell count; this is known as saturation effect (Goutelle et al. 2008; Cantrell and Cosner 2001). Saturation effect can induce bistable kinetics, which is biologically known as inoculum effect (Xu 2022; Loffredo et al. 2021; Lenhard and Bulman 2019). Importantly, it is unclear whether the simplified models of unsaturated lysing efficiency can capture the relationship among HIV viral titre, CD4+ cell count and CD8+ cell count.

To understand how saturation effect and variations in CD8+ response induced by HLA alleles determine HIV infection outcomes, a mathematical model that involves lysing infected cells induced by CD8+ cell was developed to model data from virus inhibition assays (VIA) of HIV. The model allowing CD8+ cell efficiency to saturate with an increase of both infected CD4+ count and CD8+ cell count fitted the data more robustly than unsaturated lysing models. Mathematical model with constant CD8+ cell count led to unexpectedly complex dynamical behaviours, including monostable, bistable, and even oscillatory HIV kinetics. Low lysing rate associated with non-protective HLA allele leads to monostable viral kinetic to high viral titre and oscillatory viral kinetics; at a specific interval of CD8+ cell count, HIV kinetics increase and decrease periodically. High lysing rate associated with protective HLA leads monostable viral kinetic to low viral titre and bistable viral kinetics; at a specific interval of CD8+ cell counts, small viral inoculum sizes are inhibited but not large viral inoculum sizes remain infectious. Further, CD8+ cell induced bistable kinetics exist as long as HIV infectivity remains. Finally, with CD8+ cell recruitment, HIV kinetics always exhibit oscillatory kinetics, but lysing rate is negatively correlated with range of CD8+ cell count. Overall, this analysis revealed that lysing efficiency determined by HLA alleles led to variations in within host HIV kinetics. This result implies that high lysing efficiency induced by protective HLA allele led to either maintenance or inhibition of viral infectivity depending on viral inoculum size, thereby explaining carriage of protective HLA allele does not always provide good infection results.

## Results

### Deterministic Mathematical Models of HIV Kinetics with CD8+ Cell Lysing

**Fig. 1:**
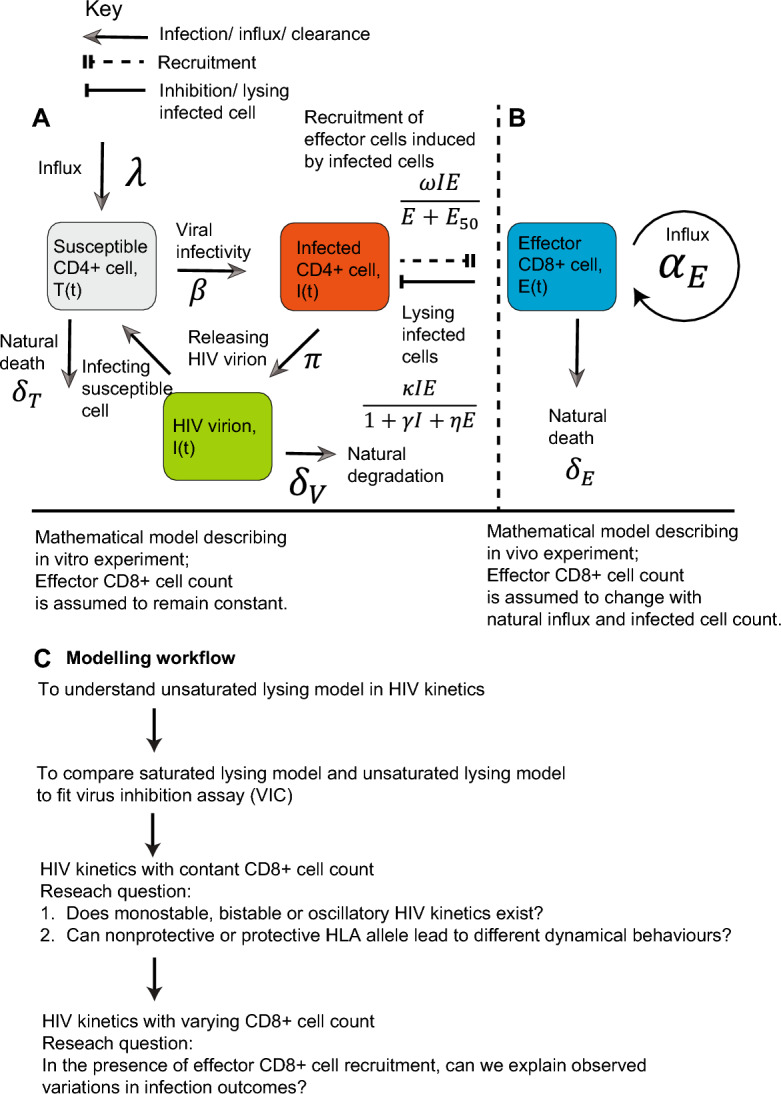
A mechanistic mathematical model of HIV kinetics in presence of CD8+ cell. Proposed mathematical models describe interaction kinetics among susceptible CD4+ cell, infected CD4+ cell and effector CD8+ cell in two scenarios, including **(A)** HIV kinetics with constant CD8+ cell count and **(B)** CD8+ cell count recruitment induced by natural influx and infected cell count. **A** Model schematic diagram: susceptible CD4+ cell proliferates at rate $\lambda$ and undergoes natural death at rate ${\delta }_{T}$. HIV virion infects susceptible cells and they become infected cell at rate $\beta$, and each infected cell release $\pi$ HIV virions. Free HIV virions naturally degrade at rate ${\delta }_{V}$. Effector CD8+ cells lyse infected cells in a saturated rate $\frac{\kappa IE}{1+\gamma I+\eta E}$, in which lysing rate of CD8+ cell increase but gradually converges to its maximum value with increase of both CD8+ cells and CD4+ cell. **B** Effector CD8+ cells are recruited to remove infected cells at rate $\omega$, but saturates when $E>{E}_{50}$. **C** The model workflow outlines the results

We designed and validated two deterministic mathematical models of HIV kinetics with unsaturated and saturated lysing efficiency. Proposed mathematical models describe interaction kinetics among susceptible CD4+ cells, infected CD4+ cells and effector CD8+ cells in two scenarios, including (A) HIV kinetics with constant CD8+ cell count (shown in System 1 and Fig. 1A) and (B) CD8+ cell count recruitment induced by infected cell count (shown in System 2 and Fig. 1B). First, susceptible CD4+ cells are naturally maintained at homeostasis with birth rate $\lambda$ and death rate ${\delta }_{T}$. They become infected with rate $\beta$ upon exposure to free HIV virion $V$, and infected cell release HIV virions at a burst size $\pi$. HIV virions naturally degrade with rate ${\delta }_{V}$. Effect CD8+ cells lyse actively infected cells with saturated rate $\frac{\kappa IE}{1+\gamma I+\eta E}$, in which lysing rate of CD8+ cell increase but gradually converges to its maximum value with increase of both CD8+ cells and CD4+ cell (shown in Fig. 1B). $\kappa$ represents CD8+ cell lysing rate, $\gamma$ and $\eta$ control the saturation (effect) in lysing efficiency as infected cells count $I$ and effector CD8+ cell count $E$ increase. In the unsaturated lysing efficiency, $\eta =\gamma =0$; namely, saturated model degenerates to unsaturated model. In HIV kinetics with CD8+ cell recruitment, effector CD8+ cell recruited at rate $\omega$ depending on the total number of infected cells, but this recruitment is limited by a saturation constant ${E}_{50}$; mathematically, CD8+ cell recruitment is described by $\omega \frac{IE}{E+{E}_{50}}$ (Reeves et al. 2020). Additionally, effector CD8+ cell naturally generate at ${\alpha }_{E}$ and die at rate ${\delta }_{E}$ (Reeves et al. 2020).

In the following analysis, model workflow outlines the results (shown in Fig. 1C). First, we re-examined role of commonly-used unsaturated lysing model in HIV kinetics. Next, we compared saturated lysing model and unsaturated lysing model to fit with HIV virus inhibition assay data. We investigated the role of saturated lysing model of CD8+ cells in determining HIV kinetics. Finally, we examined whether CD8+ cells recruitment induced by infected CD4+ cell can remain observed dynamical behaviours. Estimated HIV and CD8+ cell kinetics can explain variations in HIV infection outcomes.

For HIV kinetics with constant CD8+ cell count, we describe the rate of change of susceptible CD4+ cell, infected CD4+ cell and HIV viral titre as

1 $$\left\{ {\begin{array}{*{20}c} {\frac{dT}{{dt}} = \lambda - \delta_{T} T - \beta TV} \\ {\frac{dI}{{dt}} = \beta TV - \delta_{I} I - \frac{{\kappa I\tilde{E}}}{{1 + \gamma I + \eta \tilde{E}}}} \\ {\frac{dV}{{dt}} = \pi I - \delta_{V} V} \\ \end{array} } \right.,$$

where $T$ represents densities of susceptible CD4+ cells, $I$ represents densities of productively infected CD4+ cells, $V$ represents densities of free virus, and $\widetilde{E}$ represents constant CD8+ cell density.

For HIV kinetics with CD8+ cell recruitment, we describe the rate of change of susceptible CD4+ cell, infected CD4+ cell, HIV viral titre and effector CD8+ cell as.

2 $$\left\{ {\begin{array}{*{20}c} {\frac{dT}{{dt}} = \lambda - \delta_{T} T - \beta TV} \\ {\frac{dI}{{dt}} = \beta TV - \delta_{I} I - \frac{\kappa IE}{{1 + \gamma I + \eta E}}} \\ {\frac{dV}{{dt}} = \pi I - \delta_{V} V} \\ {\frac{dE}{{dt}} = \alpha_{E} + \omega \frac{IE}{{E + E_{50} }} - \delta_{E} E} \\ \end{array} } \right.,$$

$E$ represents CD8+ cell density with time. 3 out of 13 parameters are fit, with the remaining values fixed (see Table 1 for fixed values and Table 2 for fitted values).

**Table 1:** Summary of HIV infectivity and CD8+ T cell replication parameter

Parameter	Value	Meaning	Dimensions	References
$\lambda$	$295$	Growth rate of CD4+ susceptible cell	cells μL^−1^ day^−1^	Luo et al. (2012), Mohri et al. (2001)
${\delta }_{T}$	$0.18$	Death rate of CD4+ susceptible cell	Day^−1^	Mohri et al. (2001)
$\beta$	$3.9\times {10}^{-3}$	HIV infectivity	μL virions^−1^ day^−1^	Doitsh et al. (2010)
${\delta }_{I}$	$1.0$	Death rate of actively infected cell	Day^−1^	Markowitz et al. (2003)
$\pi$	$5.5\times {10}^{4}$	Burst size	Virions cell^−1^ day^−1^	Chen et al. (2007)
${\delta }_{V}$	$36$	Virion clearance rate	Day^−1^	Ramratnam et al. (1999)
${\alpha }_{E}$	${10}^{-5}$	CD8+ cell production rate	Cells μl − 1	Reeves et al. (2020)
${\delta }_{E}$	$0.002$	CD8+ cell removal rate	Day-1	Reeves et al. (2020)
$\omega$	$1.6$	CD8+ cell recruitment rate	μl day cell-1	Reeves et al. (2020)
${E}_{50}$	$250$	CD8+ cell limiting concentration	Cells μl	Reeves et al. (2020)
$E$	$(500-1300)$	CD8+ cell count range	Cells μl

**Table 2:** Exponential saturated lysing efficiency parameter and its corresponding dynamical behaviours

	Lysing rate $\kappa$	saturation parameter $\gamma$	Nonprotective or protective HLA	Effector CD8+ cell lysing	CD8+ cell constant or recruitment	Dynamical behaviours
A	$2.74$	$9.95$	Nonprotective	Saturated	Constant	Monostability to high viral titre
B	$2.16$	$1.13$	Nonprotective	Saturated	Constant	Oscillatory periodic cycle
C	$3.34$	$6.96$	Protective	Saturated	Constant	Bistability between low and high viral titre
D	$5.831$	$4.925$	Protective	Saturated	Constant	Monostability to ow viral titre
E	3	0	Both	Unsaturated	Constant	Monostable HIV kinetics

### HIV Kinetics with Constant CD8+ Cell and Unsaturated CD8+ Cell Lysing Efficiency Only Leads to Monostable HIV Kinetics

**Fig. 2:**
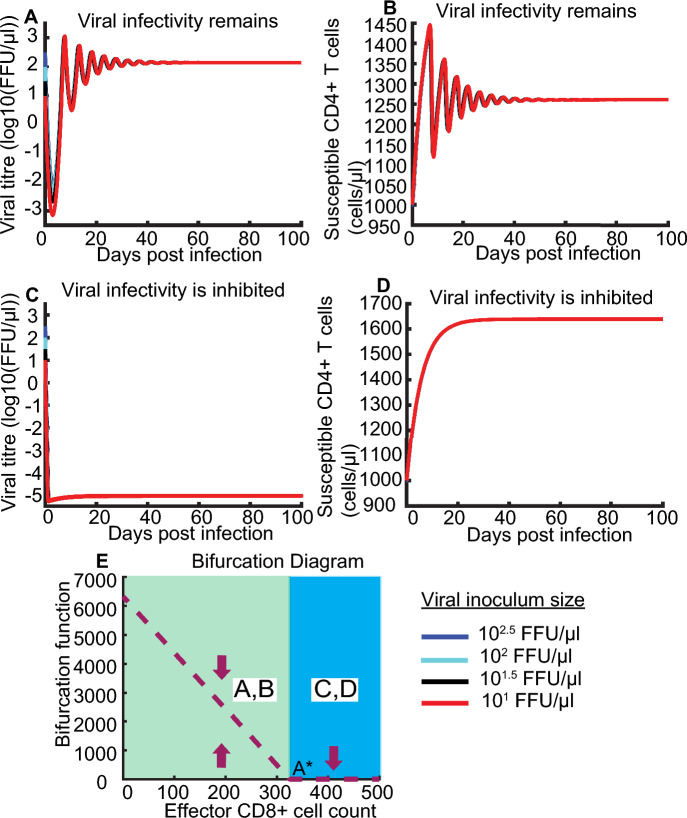
Simulated kinetics of HIV kinetics with constant CD8+ cell and unsaturated CD8+ cell lysing. **A** and** B** when CD8+ cell count is between 0 and A*, viral infectivity remains independent of viral inoculum size. **C** and **D** when CD8+ cell count is higher than A*, viral infectivity is inhibited independent of viral inoculum size. Bifurcation diagram **(E)** showing bifurcation function CD8+ cell count. The maximal capacity of viral titre decreases with increases of CD8+ cell count (brown dashed line curve). Purple arrows represent any viral inoculum size. Red, black, cyan, and blue lines represent viral kinetics with viral inoculum size 10, 10^1.5^, 10^2^ and 10^2.5^ FFU/μl in (**A**–**E**). Note that all of the lines overlap, so the cyan and black lines cannot be seen

We hypothesize that model of unsaturated CD8+ cell lysing, commonly used to quantify infected cell killing by effector or nature killer (NK) cells in vivo and in vitro (Cao et al. 2016; Ciupe et al. 2007), would only lead to monostable virus kinetics. HIV kinetics with constant CD8+ cell and unsaturated CD8+ cell lysing efficiency is given by System (1) with $\eta =\gamma =0$. Obviously, one threshold value of CD8+ cell count A* existed such that CD8+ cell count interval was divided into two regimes, shown as a bifurcation diagram (Fig. 2E). At the CD8+ cell count interval between 0 and A*, HIV infectivity remains independent of viral inoculum size (Fig. 2A, B, E). When CD8+ cell count is higher than threshold A*, HIV infectivity is inhibited independent of viral inoculum size (Fig. 2C, D, E). Qualitative analysis of HIV kinetics with constant CD8+ cell and unsaturated CD8+ cell lysing is obvious. In this case, monostable HIV kinetics cannot explain observed phenomena that carriage of protective HLA alleles may confer patients good and bad HIV control.

### CD8+ Killing Lysis Increases and Saturates with Increase of CD4+ Cell Count

Efficiency of CD8+ cell response is defined as the rate at which infected cells are killed or new infections are prevented (Elemans et al. 2012). Next, efficiency of CD8+ cells is measured by two approaches, including (1) to measure the disappearance of labelled target cells (Wick et al. 2005) and (2) to estimate the lysing by CD8+ cells from the increase in numbers of virus mutants (Elemans et al. 2012). Defect of the first approach is that efficiency of CD8+ cell is mathematically described by law of mass action (Wick et al. 2005). This induces efficiency of CD8+ cell is estimated to be very high, because it is attributable to all causes (including CD8+ cell killing, activation induced cell death, and cytopathic effect of HIV-1) as one parameter, CD8+ cell lysing rate. Efficiency of CD8+ cell killing measured by the first method is 6.5–8.4 day^−1^, and that measured by the second method is 0.1–0.2 day^−1^. Thus, 10^1.5^ time difference exists between two methods. This leads to direct question how efficiency of CD8+ T cell can be accurately estimated.

Virus inhibition assays (VIA) measures CD8+ cell-mediated inhibition of HIV replication in CD4+ cells using HIV Gag p24 intracellular staining. Number of lysed infected CD4+ cell after HIV-CD4:CD8 cell co-culture could be estimated. A total of 200μL of the $0.1\times {10}^{6}$ HIV-infected CD4+ cell and $0.2\times {10}^{6}$ CD8+ cell mixture in 5 ml round bottom FACS tubes was incubated for three days (72 h ± 3 h) at 35ºC in 5% CO_2_. Following three-day incubation, cells were then stained with antibodies to p24 antigen followed by antibodies to CD3+, CD4+ and CD8+ receptors. Samples were acquired on a Fortessa and analyzed using FlowJo (version X10.0.7r2) (Xu et al. 2021).

In existing models with infected cells and CD8+ cell interaction, efficiency of CD8+ cell lysing is assumed to be directly proportional to the product of infected cells and effector CD8+ cell by law of mass action (Wick et al. 2005; Ciupe et al. 2007). However, due to limited perforins and granzymes released/ carried by each CD8+ cell, efficiency of CD8+ cell saturates at a cell count higher than that required for lysing, for a fixed infected cell count, the rate of CD8+ cell lysing is supposed to increase and gradually converge to its maximum with increases of CD8+ cell count. To accurately estimate efficiency of CD8+ cell, we developed two models, including unsaturated model (System 3) and saturated model (System 4).

For saturated CD8+ cell lysing efficiency, we describe the rate of change of susceptible CD4+ cell, infected CD4+ cells, HIV virus and effector CD8+ cells in VIA for three-day incubation as

3 $$\left\{ {\begin{array}{*{20}l} {\frac{dT}{{dt}} = \lambda - \delta_{T} T - \beta TV} \\ {\frac{dI}{{dt}} = \beta TV - \delta_{I} I - \frac{\kappa IE}{{1 + \gamma I + \eta E}}} \\ {\frac{dV}{{dt}} = \pi I - \delta_{V} V} \\ {\frac{dE}{{dt}} = \alpha_{E} - \delta_{E} E} \\ \end{array} } \right.,$$

For unsaturated CD8+ cell lysing efficiency, we describe the rate of change of susceptible CD4+ cell, infected CD4+ cells, HIV virus and effector CD8+ cells as

4 $$\left\{ {\begin{array}{*{20}l} {\frac{dT}{{dt}} = \lambda - \delta_{T} T - \beta TV} \\ {\frac{dI}{{dt}} = \beta TV - \delta_{I} I - \kappa IE} \\ {\frac{dV}{{dt}} = \pi I - \delta_{V} V} \\ {\frac{dE}{{dt}} = \alpha_{E} - \delta_{E} E} \\ \end{array} } \right.,$$

The sum of squared error (SSE) is $SSE={\sum }_{i=1}^{n}{\left({log}_{10}\left({\text{I}}_{\text{i}}\right)-{log}_{10}F\left({I}_{i}\right)\right)}^{2}$, where ${\text{I}}_{i}$ represented experimental viral titre at time $i$. $F\left({I}_{i}\right)$ represented the estimated infected CD4+ cell count for VIA data, because one p24 focus is considered as one infected cell. Initial guesses used for parameter estimation were${\kappa }_{0}=0.1$, ${\eta }_{0}=0.001$ and${\gamma }_{0}=1$. MATLAB fmincon and GlobalSearch is used to find the global minimum in the goal region$\kappa \in [{\text{0.1,10}}^{2}]$,$\eta \in [{\text{0,10}}^{2}]$, and$\gamma \in [{\text{0,10}}^{2}]$.

**Fig. 3:**
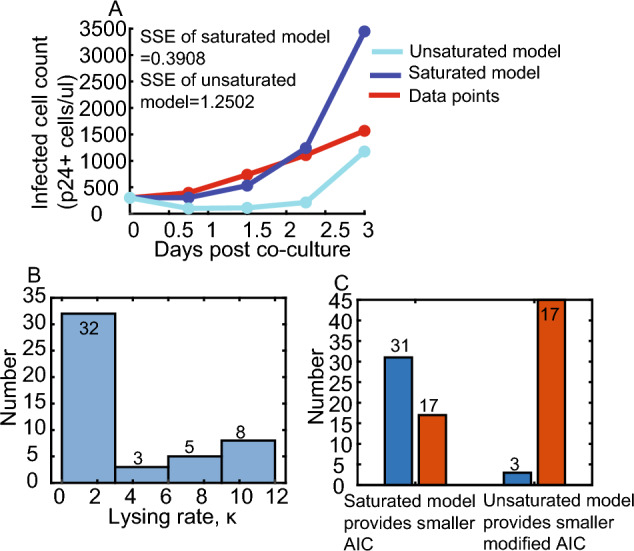
Quantitative relationship between CD8+ cell and infected cell count (p24 + cells/μl). **A** Saturated CD8+ cell lysing (light blue curve) fits to the VIA data better (red cycle) than the unsaturated CD8+ cell lysing (blue curve); sum-of-squares error (SSE) of saturated and unsaturated CD8+ cell lysing 0.3908 are 1.2502, respectively. **B** Histogram plotting CD8+ cell lysing rate $\kappa$ against its number. Majority of CD8+ cell lysing rate $\kappa$ arrange from 0 to 3. **C** Among 48 VIA datasets, saturated model provides smaller AIC, but unsaturated model provides smaller modified AIC

Saturated model better fitted VIA data estimates than unsaturated model (Fig. 3A), because saturated model provided small sum of squared error (SSE) and Akaike information criterion (AIC) (Fig. 3A). Specifically, all of SSE provided by saturated model is smaller than those provided by unsaturated model. Interestingly, 32 of 48 lysing rate $\kappa$ ranges from 0 to 3 (Fig. 3B). Moreover, because 45 of 48 saturation parameter controlling saturation effect with increase of CD8+ cell count $\eta$ is fitted to be zero, we assume that $\eta =0$ in $\frac{\kappa IE}{1+\gamma I+\eta E}$ and saturated model degrade to $\frac{\kappa IE}{1+\gamma I}$ with two parameters. However, by calculating modified AIC for small sample sizes, the modified AIC of unsaturated model were lower than that of saturated model (Fig. 3C).

### HIV Kinetics with Saturated Lysing Efficiency and Constant CD8+ Cell Count Led to Monostable, Bistable and Oscillatory Viral Kinetics

In this section, we will investigate the role of saturated lysing efficiency on HIV kinetics. CD8+ cell lysing rate corresponding to protective or non-protective HLA alleles has been used as predictor of good or bad infection outcomes, because HIV elite controllers provide increased HIV-specific CD8+ T-cell cytotoxic potential (Collins et al. 2020; Monel et al. 2019; Woldemeskel et al. 2020; Hersperger et al. 2011). Thus, we assume that protective HLA alleles provide higher lysing rate than non-protective HLA alleles do.

CD8+ cell count ranges from $100$ to $2700 \text{ cells}/\upmu \text{l}$ on initiation of cART and 10 years after cART, respectively (Helleberg et al. 2014). On initiation of cART, more than 50% of CD8+ cell distribute from $600$ to $1200 \text{ cells}/\upmu \text{l}$, particularly peak around $700 \text{ cells}/\upmu \text{l}$; 10 years after cART, more than 75% of CD8+ cell counts majorly distribute from $100$ to $500\text{ cells}/\mu\text{l}$, particularly peak around $300 \text{ cells}/\upmu \text{l}$ (Fig. 2, (Helleberg et al. 2014)). In this case, we chose median CD8+ cell count $E=300 cells/\mu l$ as example for following analysis. For other CD8+ cell counts, we can obtain similar results.

**Fig. 4:**
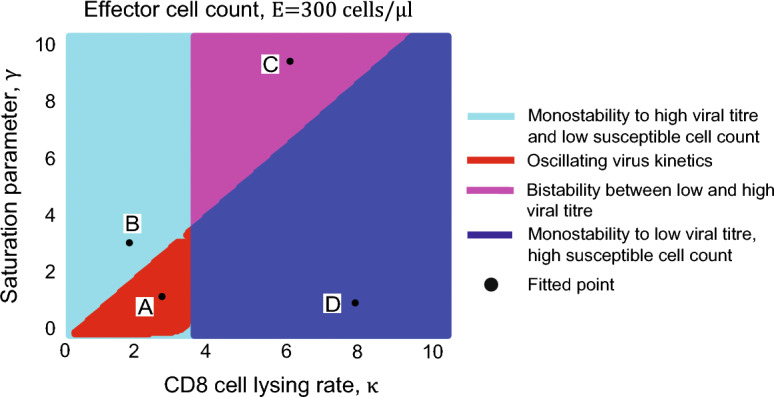
Bifurcation diagram with CD8+ cell lysing rate $\kappa$ and saturation parameter $\gamma$. System (1) exhibits four types of dynamical behaviours, including monostable kinetics to high (cyan region) or low (blue region) viral titre, bistable kinetics (purple region) between high and low viral titre and oscillating virus kinetics (red region). Black points represent fitted point, **A** ($\kappa =2.74$ and $\gamma =9.95$), **B** ($\kappa =2.16$ and $\gamma =1.13$), **C** ($\kappa =3.34$ and $\gamma =6.96$) and **D** ($\kappa =5.831$ and $\gamma =4.925$)

**Fig. 5:**
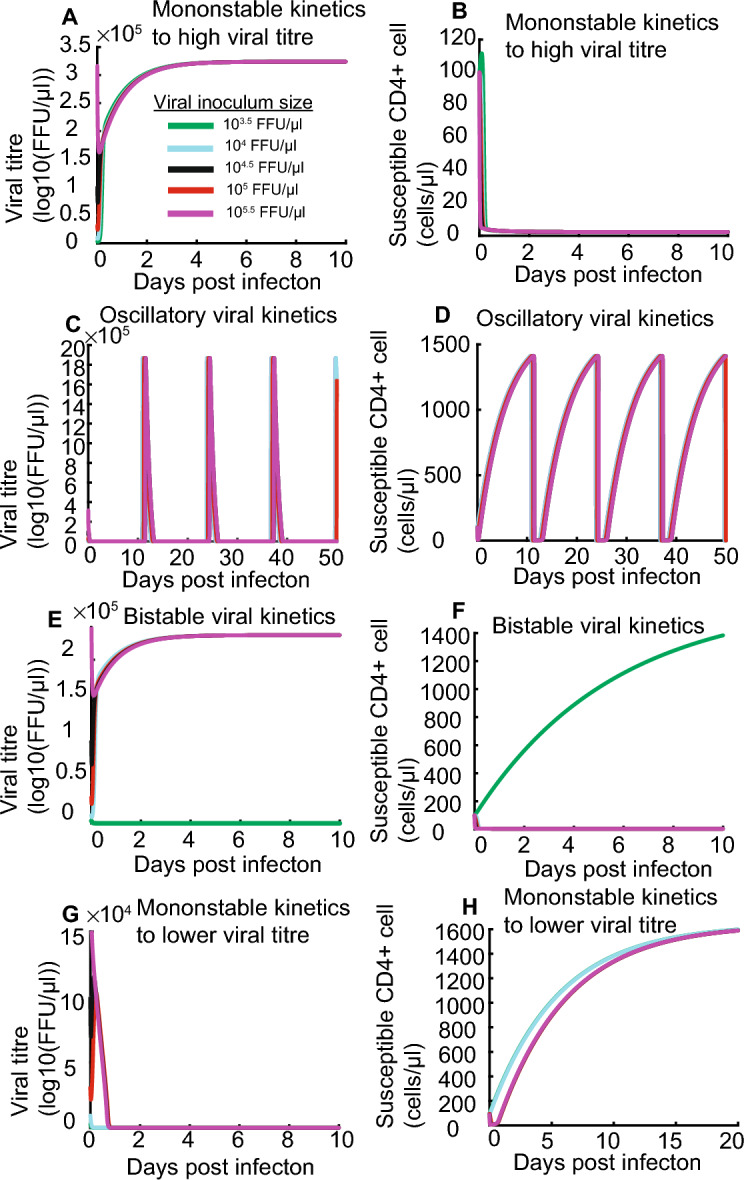
Simulated kinetics of HIV virus with constant CD8+ cell count and saturated lysing efficiency. **A** and **B** refer that low lysing rate led to high viral titre and low susceptible CD4+ cell count. **C** and **D** refers that low lysing rate and low saturation parameter led to oscillatory viral titre and susceptible CD4+ cell count. **E** and **F** refers that high lysing rate and high saturation led to bistable viral titre and susceptible CD4+ cell count, in which small viral inoculum sizes are inhibited but large viral inoculum sizes remain infectious. **G** and **H** refer that high lysing rate and low saturation parameter led to low viral titre and high susceptible CD4+ cell count. Green, cyan, black, red, and purple lines represent viral kinetics with viral inoculum sizes 10^3.5^, 10^4^, 10^4.5^, 10^5^ and 10^5.5^ FFU/μl in (**A**–**H**), for initial conditions T(0) = 10^2^ and I(0) = 10^0^. Note that some of the lines overlap, so the cyan and black lines cannot be seen

We combined the model of HIV replication kinetics (Reeves et al. 2020) with the model of saturated CD8+ cells lysing. Using saturated lysing efficiency and qualitative analysis developed in unpublished results, we found that HIV kinetics with constant CD8+ cell count exhibit unexpectedly complex dynamical behaviours (Figs. 4 and 5), including monostable viral kinetics to high or low viral titre (Figs. 4, 5A, B, G and H), oscillatory viral kinetics (Figs. 4, 5C and D) and bistable viral kinetics between high and low viral (Figs. 4, 5E and F). Specifically, monostable kinetics to high viral titre and oscillating viral kinetics coincide with low lysing rate independent of magnitude of saturation parameter (cyan and red region in Fig. 4). Bistable kinetics between low and high viral titre coincide with high lysing rate and high saturation parameter (purple region in Fig. 4), and monostable kinetics to low viral titre coincide with high lysing rate and low saturation parameter (blue region in Fig. 4).

In simulations of virus replication kinetics with saturated lysing efficiency, low lysing rate (Row A in Table 2) associated with non-protective HLA allele led to high viral titre and low susceptible cell count (shown in Fig. 5A and B). For example, at $\kappa =2.74$ and $\gamma =9.95$ in bifurcation diagram (shown in Fig. 4), viral titre converges the maximum capacity and susceptible cell count remain low with virus inoculum sizes 10^3.5^ FFU/μl, 10^4^ FFU/μl, 10^4.5^ FFU/μl, 10^5^ FFU/μl and 10^5.5^ FFU/μl. Next, low lysing rate and low saturation parameter (Row B in Table 2) induced by non-protective HLA alleles led to periodic increase and decease of viral titre and susceptible cell count (shown in Fig. 5C and D); this is triggered by saturated lysing term. For example, at $\kappa =2.16$ and $\gamma =1.13$ in bifurcation diagram (shown in Fig. 4), viral titre and susceptible cell count oscillates with post infection days independent of virus inoculum sizes, and viral infectivity maintain infectivity. Additionally, high lysing rate and high saturation parameter (Row C in Table 2) show bistable HIV viral kinetics and susceptible CD4+ cell count kinetics; namely, large viral inoculum sizes remain infectious and small viral inoculum sizes are inhibited (shown in Fig. 5E and F). This also is triggered by saturated lysing efficiency. For example, at $\kappa =3.34$ and $\gamma =6.96$ in bifurcation diagram (shown in Fig. 4), virus inoculum sizes 10^4^ FFU/μl, 10^4.5^ FFU/ μl, 10^5^ FFU/μl, 10^5.5^ FFU/μl all maintain infectivity (red, black, red and purple curve in shown in Fig. 5E), whereas inoculum size 10^3.5^ FFU/μl was inhibited (green curve in shown in Fig. 5E). Finally, high lysing rate and low saturation parameter (Row D in Table 2) led to low viral titre and high susceptible cell count (shown in Fig. 5E and F). For example, at $\kappa =5.831$ and $\gamma =4.925$ in bifurcation diagram (shown in Fig. 4), viral infectivity was efficiently inhibited independent of inoculum sizes.

**Fig. 6:**
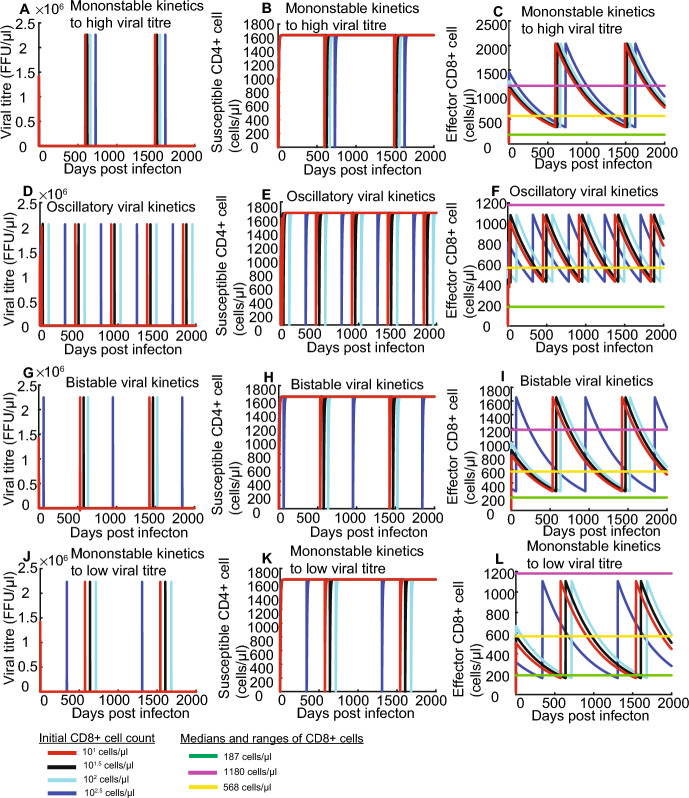
Efficiency of the CD8+ cell response corresponding to four viral kinetics types. Regardless of viral kinetics types, efficiency of the CD8+ T cell response mainly fills the gap between 0.01 and 6.5 day^−1^ in the infected cell count interval [0, 500]. **A** Efficiency of the CD8+ cell response corresponding to monostable viral kinetics to high viral titre, $\kappa =2.74$, $\gamma =9.95$, $\eta =0$, and $E=300$. **B** Efficiency of the CD8+ cell response corresponding to oscillatory viral kinetics, $\kappa =2.16$, $\gamma =1.13$, $\eta =0$, and $E=300$. **C** Efficiency of the CD8+ cell response corresponding to bistable viral kinetics, $\kappa =3.34$, $\gamma =6.96$, $\eta =0$, and $E=300$. **D** Efficiency of the CD8+ cell response corresponding to monostable viral kinetics to low viral titre, $\kappa =5.831$, $\gamma =4.925$, $\eta =0$, and $E=300$. Red and cyan line represents the upper bound and lower bound of CD8+ cell efficiency 6.5 (10^0.8129^) day^−1^ and 0.1 (10^–1^) day^−1^. Blue curve represents efficiency of the CD8+ cell response estimated by saturated cell lysing efficiency

Mathematical modelling of VIA data allows us to estimate efficiency of CD8+ cell response which are not easily and explicitly measured experimentally but determined by the data. Because frequency of HIV-1-specific CD8+ cells arrange from 2 to 15%, we chose average frequency of HIV-1-specific CD8+ cells is 7.5% (Gea-Banacloche et al. 2000) as example. In this case, the efficiency of CD8+ cell response is mathematically defined as the product of CD8+ cell count, average frequency of HIV-1-specific CD8+ T cells and saturated lysing efficiency; $EF=E\times EP\times \frac{\kappa }{1+\gamma I+\eta E}$, where $EF$ is efficiency of the CD8+ cell response, $E$ is CD8+ cell count and $EP$ is average frequency of HIV-1-specific CD8+ T cells, $\frac{\kappa }{1+\gamma I+\eta E}$ is saturated lysing efficiency. Thus, efficiency of CD8+ cell response corresponding to four viral kinetics mainly fall in the gap between low bound measuring number of virus mutants (0.1–0.2 day^−1^, (Elemans et al. 2012)) and upper boundary measuring disappearance of radio-labelled cells (6.5–8.4 day^−1^, (Wick et al. 2005)) in the infected cell count interval [0,300], including monostable viral kinetics to low or high viral titre (Fig. 6A and D), oscillatory viral kinetics (Fig. 6B) and bistable viral kinetics between high and low viral steady states(Fig. 6C).

To summarise, saturated CD8+ lysing efficiency led to monostable, oscillatory and bistable viral kinetics, and unsaturated CD8+ lysing only led to monostable viral kinetics (shown in Table 2).

### HIV Kinetics with CD8+ Cell Recruitment Induces to Oscillatory Viral Kinetics and Different Magnitude of CD8+ Cell Count

**Fig. 7:**
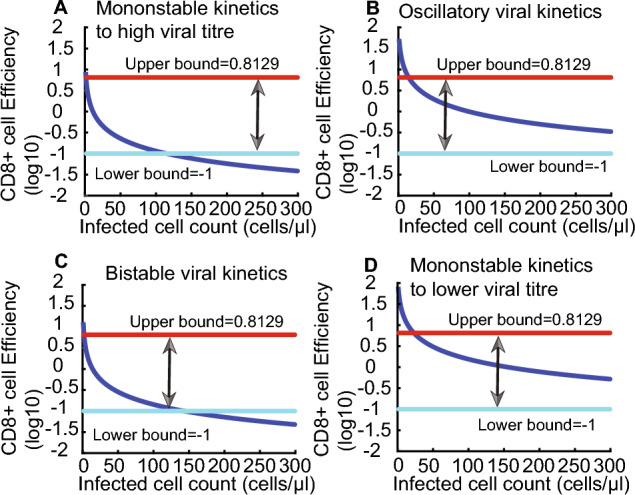
Simulated kinetics of HIV kinetics with CD8+ cell recruitment and saturated CD8+ cell lysing. **A–C** HIV viral kinetics, susceptible cell kinetics and CD8+ cell kinetics corresponds to monostable viral kinetics to high viral titre. **D–F** HIV viral kinetics, susceptible cell kinetics and CD8+ cell kinetics corresponds to oscillatory viral kinetics. **G–I** HIV viral kinetics, susceptible cell kinetics and CD8+ cell kinetics corresponds to monostable viral kinetics to low viral titre. **J, B** and **L** HIV viral kinetics, susceptible cell kinetics and effector CD8+ cell kinetics corresponds to bistable viral kinetics. Red, black, cyan and blue curve represents 10^1^, 10^1.5^, 10^2^ and 10^2.5^ cell/μl for initial conditions T(0) = 10^2^, I(0) = 0 and V(0) = 10^2^. Purple, green and yellow line represent the upper bound (1180 cell/μl), low bound (187 cell/μl) and median (568 cell/μl) of CD8+ cell count. Note that some of the lines overlap, so the cyan and black lines cannot be seen

In simulations of HIV replication kinetics with saturated lysing efficiency and CD8+ cell recruitment, HIV kinetics unexpectedly exhibit oscillatory behaviours independent of which types of HIV kinetics with constant CD8+ cell count exhibits (shown in Fig. 7). However, with CD8+ cell recruitment, viral titre provided by HIV replication kinetics with constant CD8+ cell count (delineated by System 1) always demonstrated strong negative correlation with CD8+ cell count interval (described by System 2, shown in Figs. 5 and 7).

First, monostable viral kinetics to high viral titre provided by System 1 corresponds to high CD8+ cell count level (500–2000 cells/μl) provided by System 2 above the normal CD8+ level (187–1180 cells/μl) (shown in Figs. 5A and 7C); this explains bad infection control. Next, bistable viral kinetics provided by System 1 corresponds to medium CD8+ cell interval (300–1600 cells/μl) by System 2 between normal CD8+ level (187–1180 cells/μl) (shown in Figs. 5C and 7I). Next, monostable viral kinetics to low viral titre provided by System 1 corresponds to low CD8+ cell interval (200–1200 cells/μl) by System 2 between normal CD8+ level (187–1180 cells/μl) (shown in Figs. 5G and 7L). These explains protective HLA allele can lead to both bad and good infection results. Finally, oscillatory viral kinetics provided by System 1 results in high viral titre oscillation frequency, medium susceptible cell oscillation frequency but low CD8+ cell low (400–1000 cells/μl) by System 2 between normal CD8+ level (187–1180 cells/μl) (shown in Figs. 5B and 7F).

## Discussion

Analysing HIV virus inhibition assay data for infected CD4+ cells against CD8+ cells, we shown that CD8+ cell lysing efficiency increases and gradually converges with increases of infected cell counts. Moreover, CD8+ cell lysing efficiency fall in the interval between low bound and upper boundary provided in previous results. By integrating HIV replication kinetics and CD8+ cell lysing efficiency, it was shown that HIV and CD8+ cell interaction leads to complex behaviours, including monostable, bistable and oscillatory viral kinetics, which is triggered by saturated lysing efficiency. Low lysing rate associated with non-protective HLA alleles leads to monostable viral kinetic to high viral titre and oscillatory viral kinetics. Low lysing rate associated with protective HLA alleles leads monostable viral kinetic to low viral titre and bistable viral kinetics. Further, CD8+ cell induced bistable kinetics exists as long as viral infectivity remains. Finally, with CD8+ cell recruitment, HIV kinetics always exhibit oscillatory kinetics, but lysing rate is negatively correlated with range of CD8+ cell count. Our finding provides a potential mechanistic explanation for observed good and bad HIV infection results induced by protective HLA allele. This may also explain why carriage of protective HLA alleles may not leads to good HIV infection results.

Next, efficiency of CD8+ cells is measured by two approaches, including (1) to measure the disappearance of labelled target cells and (2) to estimate the killing by CD8+ cells from the increase in numbers of virus mutants (Elemans et al. 2012). The efficiency of CD8+ cell killing measured by the first method is 6.5–8.4 day^−1^, and that measured by the second method is 0.1–0.2 day^−1^. Regardless of protective or non-protective HLA, estimated lysing efficiency fall in the gap between low bound (0.1–0.2 day^−1^, Reference (Elemans et al. 2012)) and upper boundary (6.5–8.4 day^−1^, Reference (Wick et al. 2005)), and saturated lysing efficiency provided better fitting with smaller SSE and AIC. This indicates that CD8+ cell lysing infected CD4+ cells is a continuous process with upper boundary. Interestingly, saturated parameter with effector CD8+ cell $\eta =0$ implies that saturation to upper boundary is induced by infected cell number, rather than CD8+ cell count. This grants for further research.

HIV kinetics with constant CD8+ cell count induces different viral kinetics (System 1), but HIV kinetics with constant CD8+ cell recruitment only induces oscillatory viral kinetics (System 2). System 2 considered both CD8+ cell natural generation and recruitment induced by infected CD4+ cell count. First, this qualitative change is induced by CD8+ cell recruitment with infected CD4+ cell count (mathematically, $\omega \frac{IE}{E+{E}_{50}}$ in System 2), which is positively correlated with infected CD4+ cell count. Specifically, increased CD8+ cell count suppresses infected CD4+ cell count, decreased infected CD4+ cell count brings down CD8+ cell count, and then decreased CD8+ cell count brings up infected CD4+ cell count again; this induces oscillatory infected cell and viral kinetics. Next, CD8+ cell natural generation can only lead to a constant level ($\frac{{\alpha }_{E} }{{\delta }_{E}}$) without recruitment, because rate of CD8+ cell count is independent of susceptible CD4+ cell, viral titre and infected CD8+ cell. Thus, HIV kinetics with only CD8+ cell natural generation still exhibit monostable, oscillatory and bistable viral kinetics dependant on ${\alpha }_{E}$ and ${\delta }_{E}$.

Viral titre provided by HIV kinetics with constant CD8+ cell count can predict different infection outcomes. For non-protective HLA carriers, monostable viral kinetics to high viral titre and oscillatory viral kinetics provided by System 1 correspond to high viral titre frequency and low CD8+ cell count level above the critical values provided by System 2. This indicates that estimated CD8+ cell count level cannot control HIV infectivity, and thus ART needs to remain low viral titre and normal CD4+ T cell counts. For protective HLA carrier, monostable viral kinetics to low viral titre provided by System 1 corresponds to low viral titre frequency and high CD8+ cell level below the critical values provided by System 2. This indicates that estimated CD8+ cell count level can control HIV infectivity without cART. In addition, bistable viral kinetics to low viral titre provided by System 1 corresponds to medium viral titre frequency and high CD8+ cell level between the critical values provided by System 2. This indicates that estimated CD8+ cell count level may not always control HIV infectivity.

Due to lack of HLA genotype, we made assumption that high lysing rate is associated with protective HLA alleles and low lysing rate is associated with non-protective HLA alleles. In the future research, it is worthwhile to use blood sample from non-protective and protective HLA allele to validate this assumption. Moreover, to identify the bistable CD8+ cell count interval induced by protective HLA allele and monostable CD8+ cell count interval induced by non-protective HLA allele, further work is required to experimentally validate this prediction a single experimental system.
